# Late Onset of Pseudoachalasia in Anti-Hu-Associated Syndrome

**DOI:** 10.1155/crgm/6627099

**Published:** 2025-06-16

**Authors:** Jens Jaekel, Christian Jürgensen, Frank Tacke, Christoph Jochum, Gianluca Barbone

**Affiliations:** Department of Hepatology and Gastroenterology, Charité–Universitätsmedizin Berlin, Campus Virchow-Klinikum (CVK), Campus Charité Mitte (CCM), Berlin, Germany

**Keywords:** anti-hu-associated syndrome, dysphagia, manometry, paraneoplastic, pseudoachalasia

## Abstract

**Background:** Pseudoachalasia is a rare manifestation of anti-Hu-associated syndrome. We present the case of a 61-year-old female patient presenting primarily with progressive pain and sensory disturbance of all limbs. Neurological symptoms progressed after the primary treatment response and onconeural anti-Hu-antibodies were tested positive, which is often a surrogate to paraneoplastic syndrome. Subsequently, after repeated imaging, a lung carcinoid tumor was resected without detectable recurrence after surgery. Nearly 90 months after the first neurological manifestation, the patient developed dysphagia and the diagnosis of pseudoachalasia was established by esophageal manometry. Due to recurrence after pneumatic dilatation, endoscopic botulinum toxin injection provided good clinical results for the patient.

**Purpose:** This case illustrates that anti-Hu-associated paraneoplastic pseudoachalasia may occur late in the clinical course, indicating that new-onset dysphagia in anti-Hu-positive individuals should be thoroughly investigated by imaging, endoscopy, manometry, and histology.

## 1. Background

Dysphagia and chest pain can be caused by a variety of conditions. After diagnostic work-up, only a small portion of patients are diagnosed with an esophageal motility disorder. In this group, achalasia is the most common etiology without any gender or ethnicity preference [1, 2]. A bimodal age distribution with peaks around 30 and 60 years is proposed. However, a high number of undetected cases are estimated [3]. Typical symptoms of achalasia are dysphagia, heartburn, chest pain, regurgitation, and weight loss [4]. Achalasia can be divided into primary and secondary achalasia with a blurred overlap in etiology, which was first assumed in 1919 by Sharp and described in 1947 by Ogilvie [5, 6]. Secondary achalasia, also called pseudoachalasia, accounts for around 9% of cases and is difficult to differentiate from primary achalasia [7]. Assumed nonspecific clinical features of pseudoachalasia are advanced age, rapid weight loss, short duration of symptoms, and difficulties passing the esophagogastric junction (OGJ) during endoscopy [8, 9]. Pseudoachalasia can be subclassified by its trigger in malignant and nonmalignant cases. Around 70% of pseudoachalasia has a malignant origin with a higher percentage of local tumors (OGJ) and a lower rate of distant tumors [10, 11]. In pseudoachalasia with local tumors, the infiltration leads to destruction and malfunction of plexus myentericus, which needs to be differentiated from tumor stenosis of the OGJ [12]. In a case series from 1999, around 70% of cases with pseudoachalasia were caused by an adenocarcinoma of the OGJ [13]. In distant malignancies, the destruction of the myenteric plexus is mostly caused by lymphocellular infiltration due to inflammation and consecutive fibrosis with malfunction in a paraneoplastic/autoimmune manner [12]. Helpful in differentiation to primary achalasia are onconeural antibodies, which in the case of pseudoachalasia are mainly positive for the antineuronal nuclear antibody type 1 (ANNA-1)/anti-Hu-antibody. In addition, pseudoachalasia often does not meet all inclusion criteria for achalasia in esophageal manometry with a sometimes normal integrated relaxation pressure (IRP) [9]. Without these indicators, insufficient response to pneumatic dilation therapy and a more often intact peristalsis are discussed to be diagnostic features of pseudoachalasia [9]. Nonmalignant pseudoachalasia has an even wider range of etiologies with postsurgical complications (e.g., migrated deflated laparoscopic gastric band, obstructing or kinked fundoplication, and postvagotomy), hernia, rheumatoid arthritis, Chagas disease, sarcoidosis, and others [14, 15]. Treatment options heavily depend on the individual case, but classical achalasia treatment appears to be more effective here, with regard to the re-intervention rate [16, 17]. In general, the long-term prognosis of nonmalignant pseudoachalasia is better.

In case of a local malignant tumor infiltration, surgery is the best option [17]. Without primary or secondary resectability, palliative symptomatic treatment options are limited. Botulinum toxin injection (BTI) has no indication in palliative symptomatic treatment due to its limited local and temporal efficacy. Pneumatic dilatation is a possibility but comes with an even higher perforation risk compared to paraneoplastic pseudoachalasia and primary achalasia. Self-expanding metal stents could be one option with good short-term results, but have disappointing long-term results/complications (e.g., stent migration, pain, gastroesophageal reflux, and perforation) in these advanced malignancies [18, 19]. Distant malignancies are treated according to the tumor entity and, if needed, with local endoscopic therapies. There are several case reports on pneumatic dilatation and self-expanding metal stent therapy [18, 20]. Both options need to be intensively discussed with the patient, especially due to mostly short-term relief of symptoms, high recurrence rate, and procedural complications. In the context of onconeural paraneoplastic pseudoachalasia, especially when other relevant neurologic symptoms are present, an individual interdisciplinary approach involving neurologists and immunologists should be considered (e.g., plasmapheresis and immunoglobulin administration). However, to our knowledge, no data are available for these options regarding the response of esophageal symptoms.

## 2. Case Report

### 2.1. Neurological Presentation and Work-Up

The 61-year-old female patient first presented herself at the emergency department of a local hospital in August 2012 with progressive pain and sensory disturbance of all limbs with predominant distal manifestation without further symptoms. The symptoms slowly increased over 4 weeks to unbearable intensity. Clinical examination showed hypesthesia of all four limbs and severe perturbation of sense of position with consecutive coordination disorder and sensory ataxic gait disorder. There was no paresis, but ubiquitous areflexia. Cerebrospinal fluid analysis revealed pleocytosis, elevated protein, and oligoclonal antibodies without detecting neurotropic viruses. Electrophysiological testing showed an axonal neuropathy. MRI of the head and spine, chest X-ray, and abdominal ultrasound showed no abnormalities. The diagnosis of an autoimmune-inflammatory sensory polyradiculitis in terms of a sensory Guillain–Barré syndrome was established. After the failure of corticoid treatment, immunoglobulin therapy was initiated. This led to a good clinical response, and protein levels in cerebrospinal fluid were declining. The patient was discharged. The further analysis of onconeural antibodies from the liquor revealed a strong positivity for anti-Hu antibodies.

Before further diagnostic could be performed, the patient presented herself again in the emergency department of the hospital in October 2012 with aggravation of symptoms. An intensive search for malignancy showed no positive findings (CT thorax/abdomen, esophagogastroduodenoscopy, colonoscopy, mammary ultrasound, gynecology/dermatologic examination, and FDG-PET/CT). However, now steroids and immunoglobulins resulted in no clinical improvement. After five cycles of plasmapheresis, only small improvements in neurological symptoms were seen. Therapy was changed in 2013 to rituximab. In September 2013, the patient presented in the neurological outpatient clinic of Charité for a second opinion. A pending follow-up FDG-PET/CT from the primary clinic in November 2013 showed an FDG-positive area in the lung. Surgery with lobar resection was performed with no effect on neurological symptoms. The diagnosis of an atypical lung carcinoid (pT1a pN0 (0/16) pL1 pV0 R0 G2) was made. After postsurgery recovery and rehabilitation, neurological symptoms aggravated by the end of 2014. At the beginning of 2015, again plasmapheresis and a cyclophosphamide therapy were initiated. Supporting pain medication was supervised by pain medicine specialists. Over the years of therapy until 2020, some complications such as bone fractures, alopecia, mucocele of sinus frontalis, and decompensated heart failure required further medical treatment. Unfortunately, the patient up to now suffers from neuropathic limb pain under the surveillance of pain medicine specialists and neurologists.

### 2.2. Esophageal Manifestation

**Figure 1:**
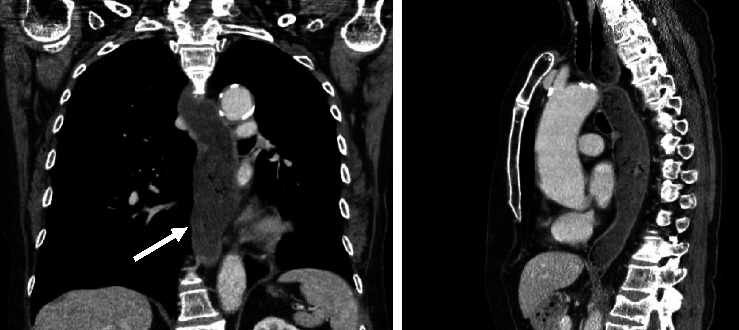
CT scan 2^nd^ of April 2020, Toshiba Aquilion 64, contrast agent Accupaque 350, and venous phase.

Dysphagia, nausea, regurgitation, and new-onset weight loss (around 9 kg in 2 months) progressively developed for 2-3 months and were first described in April 2020. The primary reason of the presentation was the suspicion of transient global amnesia. A CT-scan searching for recurring malignancy showed a distended lower esophagus over around 4 cm in diameter with fluid retention, a normal stomach, and no thickening of the esophageal wall (Figure 1, white arrow).

**Figure 2:**
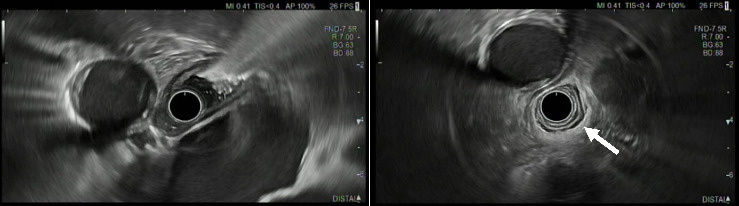
Endosonography images 7th of April 2020, Fujifilm Aloka ARIETTA 850.

We performed esophageal manometry and upper gastrointestinal endoscopy. Esophagogastroduodenoscopy showed retention of fluid and solid food, while there were no mucosal alterations and the endoscope could be easily advanced into the stomach. Endosonography showed normal esophagus wall (white arrow) stratification without suspicious lymph nodes (Figure 2).

An esophageal manometry study was performed with the ManoScan ESO (Medtronic GmbH) device. In the esophagus manometry study, the upper esophagus sphincter imaging was deferred to better visualize the lower esophagus sphincter due to a long esophagus with an OGJ at 55 cm. The study was performed with comparable gulps of water in a recumbent position with the upper body elevated 45 degrees and sitting position.

**Figure 3:**
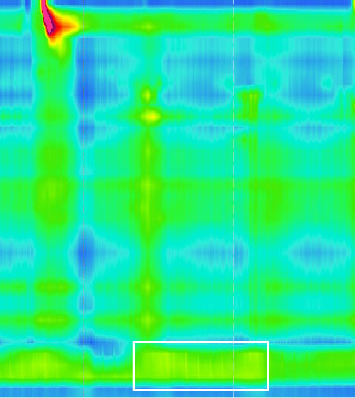
HR-manometry of the esophagus in April 2020, showing failing contractions of the whole tubular esophagus and no signs of relaxation of the lower sphincter.

Strikingly, there was no sign of relaxation (Figure 3, white box) of the lower esophagus sphincter with an elevated IRP of 23.1 mmHg (normal < 15 mmHg). Peristalsis of the proximal esophagus was concurrent and not propulsive with a pressurization of the distal esophagus. Esophageal manometry showed the picture of Type II achalasia, suspicious of pseudoachalasia due to acute onset (2-3 months), rapid weight loss, and strong elevation of anti-Hu-antibodies at that timepoint.

Based on these findings, we proceeded to endoscopic pneumatic dilatation. After the suction of the remaining fluid, the balloon was inflated for 2 min to 30 mm. No perforation, but small mucosal bleedings were detected afterward. The patient experienced an immediate improvement in symptoms and was discharged with a recommendation to return when symptoms would aggravate again. In September 2020, the patient presented herself with returning symptoms for 3-4 weeks primarily with regurgitation. Endoscopy showed an easy passage into the stomach with an erythematosus, ulcerative gastritis and negative helicobacter test upon histology. The dilatation therapy with a 30 mm balloon for 2 min was repeated. After discharge, the patient had to fight the progress of the limb pain/paresthesia with different treatment regimens under the supervision of the department of pain medicine over the next years. The last presentation was in February 2023 in the emergency department with nausea without vomiting, progressive dysphagia, and weight loss over the last weeks. Gastroscopy showed a gaping lower esophagus sphincter around 5 mm with easy passage of the gastroscope and no mechanical obstruction. A few days later esophageal manometry was performed in a 45-degree seated position with 10 fluid test gulps and a solid test meal.

**Figure 4:**
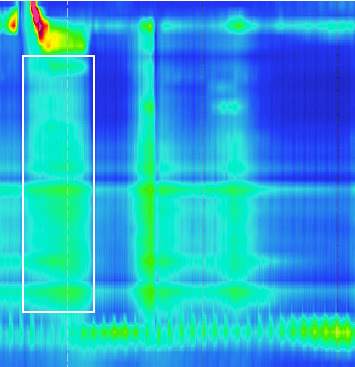
HR-manometry of the esophagus in February 2023, test with fluid, showing pathological contractions of the tubular esophagus with the relaxation of the lower sphincter.

**Figure 5:**
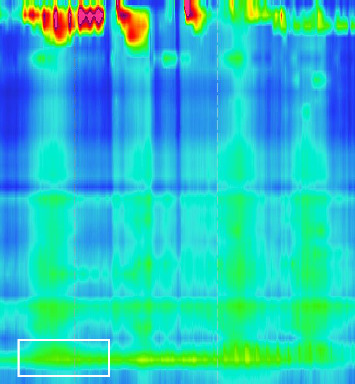
HR-manometry of the esophagus in February 2023, test with food, showing failing relaxation of the lower sphincter together with pathological contractions of the esophagus.

Lower esophagus sphincter relaxation with fluids was on time, complete and IRP was normal. The tubular esophagus showed no sign of effective transport (Figure 4, white box). With the solid food test meal, the lower esophagus sphincter showed no relaxation compared to fluid tests (Figure 5, white box). Thus, the study showed ineffective esophagus motility with the picture of a Type II achalasia to solid food. Dietetic recommendations with smaller food portions, within return higher frequency of food intake, did only improve the symptoms a little bit. Thus, we subjected the patient to endoscopic BTIs. During a gastroscopy, 25 I.E. botulinum toxin was injected into every quadrant of the cardia. The patient showed on the following day an improvement and could be discharged. Within the next 12 months and to our knowledge up to now, no more interventions were needed.

## 3. Discussion

We herein report the case of a patient who presented initially with peripheral neurological symptoms and positive onconeural anti-Hu antibodies due to an atypical carcinoid of the lung. Years later, dysphagia developed with rapid weight loss, nausea, and regurgitation. After the exclusion of carcinoid recurrence and a new adenocarcinoma of the OGJ, an esophageal manometry was performed. Most notably there was no sign of relaxation of the lower esophagus sphincter with elevated IRP and concomitantly abnormal distal esophagus motility. The manometric assessment was suggestive of achalasia Type II, but fitting context factors (advanced age, fast weight loss, short symptom duration, and ongoing strongly positive anti-Hu antibodies) led to the diagnosis of pseudoachalasia. A destruction of the myenteric plexus of the esophagus by lymphocellular infiltration as part of an anti-Hu syndrome is assumed to be the potential cause of the pseudoachalasia. This case shows the need for a multidisciplinary approach to paraneoplastic anti-Hu-associated syndrome with the involvement of different organs.

In this case, coincidental with new gastrointestinal symptoms, also, new neurological symptoms developed and the pre-existing polyneuropathic pain aggravated without any sign of tumor recurrence. Opposite to previously published cases, in which gastrointestinal motility disorder due to paraneoplastic syndrome preceded the diagnosis of a malignant tumor, in our patient, dysphagic symptoms due to pseudoachalasia developed late in the course of the malignant disease around 90 months after the first neurologic symptoms [12, 21]. A possible relation between late onset of gastrointestinal symptoms due to suspected paraneoplastic inflammatory reaction and early immunomodulating therapy for neuropathic pain can not be further clarified. According to Rossi et al., around 30% of the patients with anti-Hu-associated syndrome develop gastrointestinal symptoms [22]. 37.5% of these patients reported an improvement in symptoms after immunomodulation and/or chemotherapy. Isolated gastrointestinal symptoms of one segment of the gastrointestinal tract, e.g., of the esophagus such as in our case in paraneoplastic anti-Hu-associated dysmotility can be up to 40% of patients [22]. Another possibility in our case is the coincidental development of a paraneoplastic sensory neuropathy of the limbs and an accompanying achalasia Type II. In the present case, many indicators (only minor IRP elevation, advanced age, rapid weight loss in 2 months, short duration of symptoms for around 2-3 months, strongly elevated anti-Hu antibodies at the timepoint of esophagus manometry, and parallel aggravation of neuropathic symptoms) pointed to secondary achalasia after detecting the pattern of achalasia Type II in the esophageal manometry [23]. The manometric report showed an elevated IRP, which is not always the case in pseudoachalasia and acts as a measurable surrogate for an OGJ motility disorder. Second, there was a simultaneous panesophageal pressurization without signs of propulsive bolus transport, as a surrogate for a panesophageal motility disorder. The assumption to cure all symptoms with a distal esophageal intervention was unlikely, so the rationale was to choose the least harmful intervention with good symptom relief. The patient already suffered from a reduced quality of life due to constant neural limb pain, so one argument for an intervention was to reduce the probability of additional reduction in quality of life.

The decision for pneumatic dilatation was made after an intensive discussion with the patient explaining different therapy options. An alternative treatment option to pneumatic dilatation would be peroral endoscopic myotomy (POEM), which offers a higher long-term success rate compared to pneumatic dilation according to a multicentric randomized controlled trial from 2019 in primary achalasia [24]. With an increased risk for reflux symptoms after POEM and therefore reduction in quality of life, the decision was made for pneumatic dilatation. However, there are no comparative data on pseudoachalasia for any of the interventions. In pseudoachalasia, one major problem is establishing the correct diagnosis. Around a quarter of the patients are misdiagnosed and are accordingly mostly treated as achalasia [15]. After all, most pseudoachalasia entities are related to local tumor infiltration leaving conventional therapeutic options (surgery, chemotherapy, and radiotherapy) for the few patients detected early and palliative endoscopic solutions (e.g., covered metal stents and, individual solutions) with overall mixed results for late discovery [18]. In our case with pseudoachalasia due to a distant tumor, paraneoplastic syndrome, and new esophageal symptoms without signs of tumor relapse, classical achalasia therapy or palliative endoscopic therapy are therapeutic options. Immunomodulating therapy has no value due to poor outcomes in paraneoplastic settings [22]. Typically, BTI therapy has also no therapeutic role in pseudoachalasia therapy due to mostly local tumor infiltration and its reduced local and temporal efficacy [17]. This case showed after two pneumatic dilatations an easy passage in the stomach with an endoscope, no local tumor manifestation, lost relaxation due to solid food, and paradoxical contractions of the lower esophagus sphincter after contraction of the upper esophagus sphincter in esophagus manometry, suggesting a complex motility disorder due to paraneoplastic syndrome. The evaluation of the case in the department led to the decision that another pneumatic dilatation had an inferior chance of symptom release. Therefore, BTI therapy was initiated as a symptom-focused treatment approach, in agreement with the patient. After the intervention, there was a symptom improvement. This decision cannot be directly transferred to other cases due to an individual approach.

Pseudoachalasia is still lacking comparative studies on different treatment options. This leaves physicians and patients to decide which might be the best therapy in the individual case derived from achalasia data. In our case, manometric findings led the course of treatment, ultimately ensuring improvement in symptoms and quality of life for the patient.

## Data Availability

The data that support the findings of this study are available on request from the corresponding author. The data are not publicly available due to privacy or ethical restrictions.
